# Perceptual Threshold Level for the Tactile Stimulation and Response Features of ERD/ERS-Based Specific Indices Upon Changes in High-Frequency Vibrations

**DOI:** 10.3389/fnhum.2017.00207

**Published:** 2017-04-25

**Authors:** Mi-Hyun Choi, Boseong Kim, Hyung-Sik Kim, Seon-Young Gim, Woo-Ram Kim, Soon-Cheol Chung

**Affiliations:** ^1^Department of Biomedical Engineering, Research Institute of Biomedical Engineering, College of Biomedical and Health Science, Konkuk UniversityChungju, South Korea; ^2^Department of Philosophical Counseling and Psychology, Dong-Eui UniversityBusan, South Korea

**Keywords:** event-related desynchronization (ERD) response, event-related synchronization (ERS) response, high-frequency vibrotactile stimulation, perceptual threshold level, specific indices based on ERD/ERS

## Abstract

This study was conducted to identify characteristics of the perceptual threshold level and electroencephalogram (EEG) responses to vibrotactile stimulations at various high frequencies, and to examine the possibility of distinguishing vibrotactile stimulations by frequency through such response characteristics. The vibrotactile stimulations of six frequencies (150, 200, 225, 250, 275 and 300 Hz) were exerted on the first joint of the right index finger. The perceptual threshold level was defined as the first minimum perceived intensity when the intensity stimulation was exerted step by step at each vibration frequency. EEG response characteristics were investigated by examining a single index corresponding to the peak or area of event-related desynchronization/synchronization (ERD/ERS) and seven specific indices derived by combining the single ERD/ERS indices. There was a significant difference in the perceptual threshold level across different frequencies. Specifically, the differences in vibration stimulus between 150 Hz and 200 Hz, and between 150 Hz and 225 Hz were significant. Of the EEG response characteristics, the single index of the peak or area of ERD/ERS did not show a significant difference by frequency. However, (ERS−ERD), ERD × (ERS−ERD), and ERS × (ERS−ERD) showed a significant difference between vibration stimulations at 150 Hz and 200 Hz, and between vibration stimulations at 150 Hz and 225 Hz, among the specific indices combined using the peak values of ERD/ERS. Furthermore, ERS × (ERS−ERD) showed a significant difference between 150 Hz and 225 Hz, and between 225 Hz and 275 Hz among the specific indices combined using the area of ERD/ERS. The perceptual threshold level and the specific indices of ERD/ERS suggested in the present study can be used as quantitative measurement indices to distinguish high-frequency vibration stimulation.

## Introduction

Tactile stimulation is utilized in many areas involving the development of haptic systems, as well as being applied to the existing interface centered on vision and audition. The most common method for applying tactile information has involved vibration. Although tactile information can generally be divided into vibration, pressure, heating and others (Dyck et al., 1978), the application of vibrotactile information is preferred due to facilitated device utility; easily applicable devices mainly use vibrotactile information (Kim et al., 2010; Choi and Kuchenbecker, 2013). When considering the vast distribution and features of receptors used by humans to process tactile information, vibrotactile information is most closely related to Meissner’s corpuscles and Pacinian corpuscles (Johansson and Vallbo, 1979; Bark et al., 2008). To utilize in devices considering the features of tactile information processing, vibrotactile information should be clearly distinguished in a quantitative fashion. Accordingly, recent work has used electroencephalogram (EEG) features to detect quantitative distinctions of vibrotactile information.

The EEG data commonly used for the quantitative exploration of movement are called event-related desynchronization/synchronization (ERD/ERS); this represents the response to certain frequencies in the somatic domain of the brain (Pfurtscheller et al., 1997). Specifically, ERD is defined as a signal decrease in the alpha and beta frequency bands as a result of external movement stimulation, whereas ERS refers to a signal increase in the alpha and beta frequency bands as a result of the stimulation (Pfurtscheller and Lopes da Silva, 1999b; Houdayer et al., 2012; Bulea et al., 2014). Because ERD and ERS are indicators of sensitivity to physical movement, they are considered to be very important in distinguishing frequency-based vibrotactile information based on EEG (Yuan et al., 2010; Jeon et al., 2011).

Several studies have examined ERD and ERS features for vibrotactile stimulation. For example, Spitzer et al. (2010) generated vibrotactile stimulation at a frequency of 16–41 Hz for the index finger, which can be perceived as flutter, to examine the ERD/ERS features. Neuper and Pfrurtscheller (2001) also considered ERD/ERS features by generating a vibrotactile stimulation at a frequency of less than 22 Hz on the hand and foot. These two studies shared some commonalities. First, vibrotactile stimulations were classified by controlling the frequency, which is the most fundamental physical trait of vibration. In addition, no stimulation more intense than a “flutter” was considered from a qualitative perspective, even though the physical classification is available for low- and high-frequency vibrotactile stimulation. Although examining the use of an EEG index for classification of very minute vibrations is important, the disadvantage is low applicability for devices requiring stimulation that can be qualitatively distinguished at the human perception level. With regard to EEG measurements, another disadvantage is that only a single index of ERD and ERS is used. Although ERD and ERS are sensitive to physical movement, in order to ensure that vibrotactile information is clearly distinguished and perceived, it is necessary to add specific indices that can amplify the excitatory and inhibitory effects of ERD/ERS of high-frequency vibration stimulations as well as low-frequency vibration stimulations.

Therefore, it would be possible to distinguish the high-frequency vibrotactile stimulations by frequency using more specific indices that amplify the excitatory and inhibitory effects of ERD/ERS, as well as the basic information of ERD/ERS as EEG indices sensitive to physical movement due to the nature of vibrotactile stimulations. An effective approach to vibrotactile stimulation is to utilize frequencies that can be qualitatively distinguished at the human perceptual level. This study thus examined the features of seven ERD/ERS indices segmented with vibrotactile stimulations of relatively high frequencies (150–300 Hz) compared to preceding studies in order to determine whether or not these indices can be used for detailed classification of vibrotactile stimulation.

## Experimental Method

### Subjects

A total of 18 healthy male adults (age: 22.9 ± 3.5 years) with normal perceptual and cognitive functions participated in this experiment. Subjects with physical and mental problems or who were receiving medication were excluded from the experiment. Before the experiment, we requested that participants not use tobacco, caffeine, drugs, or alcohol for a week prior to the experiment because these substances are known to affect the central nervous system. On the day of the experiment, we excluded participants who failed to follow this restriction. All subjects were found to be right-handed according to the revised Edinburgh Reading Test (Oldfield, 1971). This study was carried out in accordance with the recommendations of the Institutional Review Committee of Konkuk University, and written informed consent was obtained from all subjects in accordance with the Declaration of Helsinki (KU-IRB-11-46-A-1).

### Vibrating Stimulator

**Figure 1:**
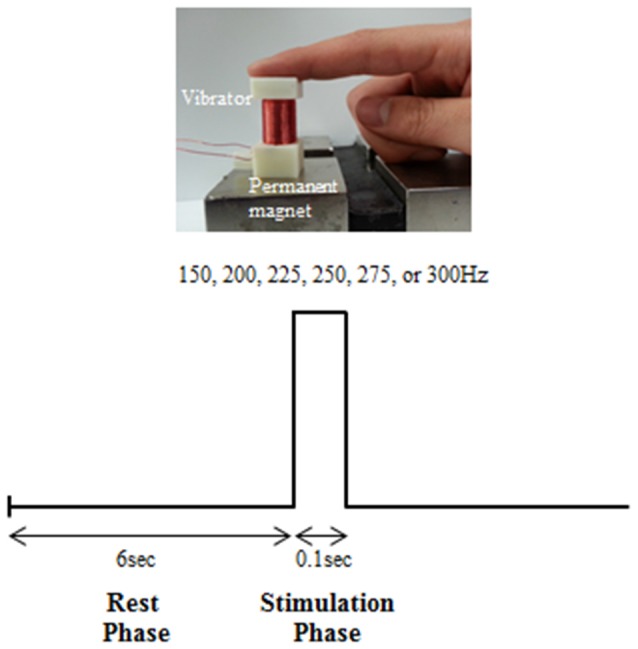
**Experimental design**.

A vibrating stimulation was exerted with the use of a vibration stimulation system developed by our research team (Chung et al., 2014). The developed system generates vibrations by sending a sinusoidal electric signal to the solenoid coil and using a Lorentz force that is electromagnetically generated by interaction with an external magnetic field (Figure 1). The stimulation frequency could be controlled within a range of 0–400 Hz. The stimulation intensity could be controlled in 20 stages. Stimulation was controlled with the use of E-Prime S/W (Psychology Software Tools, Inc., Sharpsburg, PA, USA) installed on a PC. The area of contact between the solenoid coil and finger was 1.8 × 1.8 cm^2^.

### Measurement of the Perceptual Threshold Level

Before the experiment to measure the EEG, we examined the perceptual threshold level for different intensities of six vibration frequencies: 150, 200, 225, 250, 275 and 300 Hz. The perceptual threshold level was defined as the stimulation at the minimum intensity that the subject perceived for each vibration frequency. The experimental trial to examine perceptual threshold level comprised a rest phase (6 s) and stimulation phase (0.1 s; Figure 1). In the rest phase, the participant was in a stable, relaxed condition without any stimulus being applied. In the stimulation phase, a certain stimulation intensity was exerted at a certain vibration frequency to the first joint of the right index finger of the subject. The vibration frequency (150, 200, 225, 250, 275 and 300 Hz) and intensity (level 1–20) were exerted in sequence, and the level of stimulation intensity at which the subject felt the vibration for each frequency was determined.

In order to verify the significant difference in perceptual threshold level obtained for each frequency, a repeated one-way analysis of variance (ANOVA; PASW Statics 18) was conducted. Bonferroni analysis was carried out for *post hoc* verification.

### Experimental Design to Measure EEG

The experimental trial to measure the EEG also comprised a rest phase (6 s) and stimulation phase (0.1 s; Figure 1). The rest phase was the state in which no vibration stimulation was exerted. In the stimulation phase, different intensities of six vibration stimulations (150, 200, 225, 250, 275 and 300 Hz) were exerted on the first joint of the right index finger. A total of 60 trials were conducted by randomly repeating the stimulation of six vibration frequencies 10 times. This was defined as one session. Such sessions were conducted three times, and there was a 10–min break between each session. Overall, 30 stimulations were exerted at each vibration frequency. Level 10 (1.5 gravity), which was a stimulation intensity that could be perceived at all frequencies based on the results of the initial perceptual threshold experiment, was used as the stimulation intensity for the EEG experiment.

### Electroencephalogram (EEG) Measurement

Neural signals were recorded using an EEG device (Quick Amp, Brain Product, Inc., Morrisville, NC, USA) during the experiment. EEGs were measured at a sampling frequency of 500 Hz. Based on the 10–20 electrode system, Ag/AgCl electrodes were placed over a total of 16 locations (AF3/4, F3/4/z, FC1/2/5/6, C3/4/z, CP1/2, Pz, Oz) including C3, which is the somatic sensory domain (Somatosensory area in the left hemisphere). In order to restrict stimulations other than vibration (e.g., visual and auditory), external visual factors were blocked with the use of a black partition. Each subject was instructed to place his arm on the desk with a vibration stimulator while in a comfortable sitting position on a chair. The experiment started when the EEG was stable with limited body movement.

### Data Analysis

Acquired data were analyzed with MATLAB (Mathworks, Novi, MI, USA). Bandpass filtering was conducted at 0.5–40 Hz after channel location of the acquired data. Because the vibration stimulation was exerted on the right index finger, detailed analysis was conducted regarding the somatic sensory domain of the contralateral side (i.e., at location C3). The recorded EEG signals were filtered using a Butterworth filter (0.1–50 Hz). The data of one participant was discarded because of abnormal impedance range. Once the EEG had been divided into epochs from 0.5 s before stimulation to 3 s after stimulation for each vibration frequency and short-time Fourier transform (sTFT) was conducted for spectrum analysis over a total period of 3.5 s. The sTFT data were divided into three frequency bands: the alpha domain (10–13 Hz), beta domain (20–27 Hz), and sensorimotor rhythm (SMR) domain (10–30 Hz). Then, the power was calculated. The power was defined as a dB value, which was subtracted from the log after squaring the sTFT value. After all trials of each subject were averaged, the data from all subjects were averaged to calculate the average power.

By calculating the average power value for the three frequency domains, the peak and area of ERD and ERS were extracted. The peak and area were extracted by comparing the power values before stimulation (0 s) to 1 s after stimulation for ERD and between 1 s and 3 s after stimulation for ERS. Along with the peak and area of ERD and ERS for the three frequency domains, the following seven indices were also calculated: ERS − ERD, ERS + ERD, ERS/(ERS − ERD), ERD/(ERS − ERD), ERS × (ERS − ERD), ERD × (ERS − ERD), and (ERS + ERD)/(ERS − ERD). These seven indices were calculated from the peak and area of ERD and ERS in the alpha domain (10–13 Hz), and the same calculation was conducted in the beta and SMR domain. Based on a reference that suggested a significant change in the ERD signal in the alpha domain and ERS signal in the beta domain due to tactile stimulation, the seven indices were each calculated using ERD (peak and area) in the alpha domain and ERS (peak and area) in the beta domain.

Reliability analysis was conducted to verify the reliabilities of the peak and area values of both ERD/ERS extracted from the trials of each subject and the seven indices to which ERD/ERS were applied. The results showed that all values of Cronbach’s alpha, a statistical measure of reliability, were over 0.8, which indicates a high level of reliability. Accordingly, the following analysis was performed.

In order to verify whether or not there was a significant difference in vibration frequencies measured by the seven indices and the peak and area of ERD and ERS, a one-way repeated ANOVA (PASW Statistics 18) was conducted, and Bonferroni analysis was conducted for *post hoc* verification. Additionally, linear-by-linear association was used to examine the association between perceptual threshold level and each index value.

## Results

**Figure 2:**
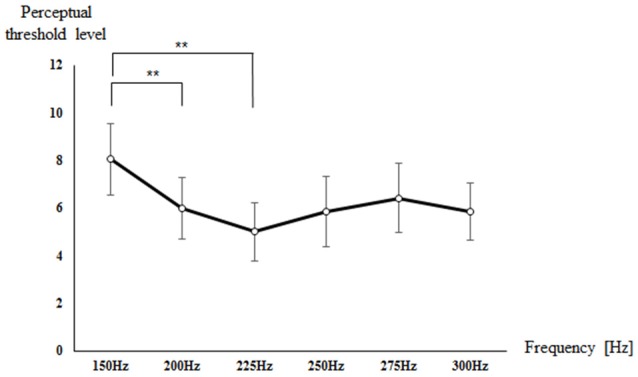
**Mean value of the perceptual threshold level of all subjects for each frequency**. **p < 0.01.

Figure 2 shows the perceptual threshold level for each frequency. The mean and standard deviation (SD) of the perceptual threshold level was 8.05 ± 2.99 at 150 Hz, 6.00 ± 2.58 at 200 Hz, 5.00 ± 2.47 at 225 Hz, 5.84 ± 2.93 at 250 Hz, 6.42 ± 2.93 at 275 Hz, and 5.84 ± 2.39 at 300 Hz. There was a significant difference in the perceptual threshold level for each frequency (*p* < 0.001). The *post hoc* verification showed a significant difference in perceptual threshold level between 150 Hz and 200 Hz (*p* = 0.002) and between 150 Hz and 225 Hz (*p* = 0.006). In other words, the perceptual threshold level was lower at 200 and 225 Hz compared to at 150 Hz, which indicates high sensitivity to the vibrotactile frequency.

**Figure 3:**
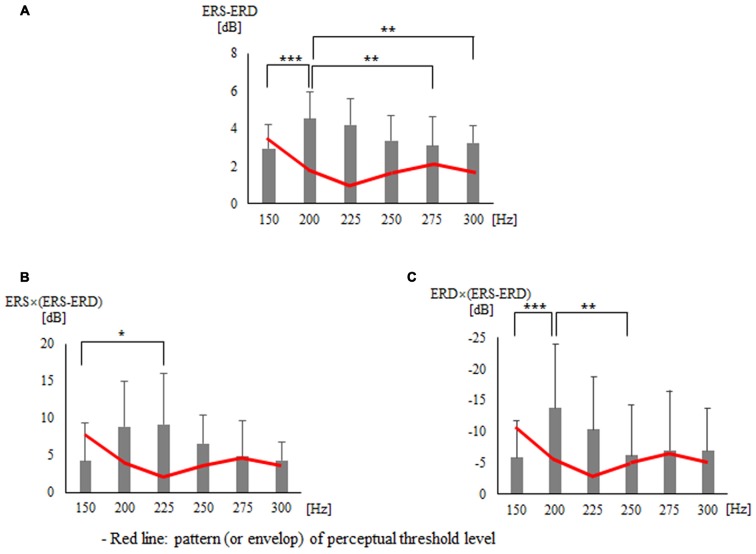
**Findings for three types of indices that presented significant differences in statistical analysis using the peak of the event-related desynchronization (ERD) and event-related synchronization (ERS): (A)** ERS − ERD** (B)** ERS × (ERS − ERD)** (C)** ERD × (ERS − ERD). *p < 0.05, **p < 0.01, ***p < 0.001.

The three frequency domains of alpha (10–13 Hz), beta (20–27 Hz), and SMR (10–30 Hz) showed no significant difference between all vibration frequencies in terms of not only the peak and area of ERD and ERS but also the additional seven indices. Tables 1, 2 present the findings for the seven indices calculated with peak and area of ERD in the alpha domain and ERS in the beta domain. First, the results of the statistical analysis of indices using the peak of ERD and ERS showed a significant difference for each vibration frequency with ERS − ERD (*p* = 0.001), ERS × (ERS − ERD; *p* = 0.006), and ERD × (ERS − ERD; *p* = 0.012; Table 1 and Figure 3). The *post hoc* verification revealed a significant difference between 150 Hz and 200 Hz (*p* < 0.001), 200 Hz and 275 Hz (*p* = 0.009), and 200 Hz and 300 Hz (*p* = 0.006) for ERS − ERD (Figure 3A). ERS × (ERS − ERD) showed a significant difference between 150 Hz and 225 Hz (*p* = 0.047; Figure 3B). ERD × (ERS − ERD) showed a significant difference between 150 Hz and 200 Hz (*p* < 0.001) and between 200 Hz and 250 Hz (*p* = 0.004; Figure 3C). In other words, the above three index values were higher for vibration frequencies of 200 Hz and 225 Hz compared to at 150 Hz. The analyses of the association of perceptual threshold level with the three indices that showed a significant difference revealed that there was a significant difference in ERS − ERD (*p* = 0.047) and ERS × (ERS − ERD; *p* = 0.031), whereas no significant difference was observed in ERD × (ERS − ERD; *p* = 0.100).

**Table 1:** **Findings for seven types of indices calculated with the peak of the event-related desynchronization (ERD) in the alpha domain and event-related synchronization (ERS) in the beta domain**.

	ERD peak	ERS peak	ERS−ERD (peak to peak)	ERS + ERD	ERS/(ERS−ERD)	ERD/(ERS−ERD)	ERS × (ERS−ERD)	ERD × (ERS−ERD)	(ERS + ERD)/(ERS−ERD)
150 Hz	−1.70 ± 1.09	1.25 ± 0.88	2.95 ± 1.25	−0.46 ± 1.54	0.46 ± 0.28	−0.54 ± 0.28	4.21 ± 5.19	−5.95 ± 4.93	−0.08 ± 0.56
200 Hz	−2.73 ± 1.36	1.81 ± 1.04	4.54 ± 1.43	−0.92 ± 1.95	0.41 ± 0.21	−0.59 ± 0.21	8.83 ± 6.15	−13.74 ± 9.21	−0.17 ± 0.42
225 Hz	−2.19 ± 1.19	2.01 ± 1.00	4.20 ± 1.39	−0.18 ± 1.71	0.49 ± 0.20	−0.51 ± 0.20	9.18 ± 6.88	−10.32 ± 8.74	−0.03 ± 0.40
250 Hz	−1.50 ± 1.23	1.85 ± 0.79	3.35 ± 1.32	0.36 ± 1.59	0.62 ± 0.31	−0.38 ± 0.31	6.62 ± 3.79	−6.25 ± 5.9	0.24 ± 0.63
275 Hz	−1.67 ± 1.51	1.43 ± 0.90	3.11 ± 1.50	−0.24 ± 1.97	0.54 ± 0.32	−0.46 ± 0.32	4.84 ± 4.85	−6.95 ± 8.19	0.09 ± 0.63
300 Hz	−1.83 ± 1.42	1.41 ± 0.75	3.24 ± 0.91	−0.42 ± 2.08	0.52 ± 0.38	−0.48 ± 0.38	4.29 ± 2.51	−6.99 ± 6.22	0.03 ± 0.75
*p* value	0.076	0.064	0.001	0.453	0.369	0.369	0.006	0.012	0.369

**Table 2:** **Findings for seven types of indices calculated with the area of the ERD in the alpha domain and ERS in the beta domain**.

	ERD area	ERS area	ERS−ERD	ERS + ERD	ERS/(ERS−ERD)	ERD/(ERS−ERD)	ERS × (ERS−ERD)	ERD × (ERS−ERD)	(ERS + ERD)/(ERS−ERD)
150 Hz	−34.14 ± 28.38	45.15 ± 47.57	77.39 ± 56.27	12.91 ± 54.82	0.55 ± 0.33	−0.45 ± 0.33	5669.91 ± 10006.89	−3505.24 ± 3799.05	0.10 ± 0.67
200 Hz	−55.80 ± 36.22	67.64 ± 63.95	119.68 ± 62.73	8.09 ± 83.04	0.51 ± 0.27	−0.49 ± 0.27	11458.94 ± 15219.16	−7217.54 ± 6328.77	0.02 ± 0.55
225 Hz	−39.19 ± 34.15	83.50 ± 63.81	122.69 ± 64.75	44.32 ± 79.27	0.66 ± 0.25	−0.34 ± 0.25	13596.51 ± 18913.66	−5415.39 ± 6572.15	0.31 ± 0.51
250 Hz	−34.86 ± 20.29	78.54 ± 44.72	105.65 ± 46.89	51.43 ± 53.62	0.67 ± 0.24	−0.33 ± 0.24	10026.32 ± 8506.17	−4129.12 ± 2954.68	0.33 ± 0.47
275 Hz	−34.40 ± 37.09	45.27 ± 44.30	75.85 ± 51.64	14.69 ± 62.72	0.55 ± 0.32	−0.45 ± 0.32	4988.39 ± 8093.22	−3693.58 ± 4900.62	0.10 ± 0.65
300 Hz	−39.03 ± 32.94	63.36 ± 46.27	94.53 ± 39.98	25.14 ± 71.5	0.61 ± 0.32	−0.39 ± 0.32	7348.88 ± 8676.18	−3942.92 ± 3749.22	0.21 ± 0.63
*p* value	0.344	0.138	0.033	0.234	0.575	0.575	0.031	0.279	0.575

**Figure 4:**
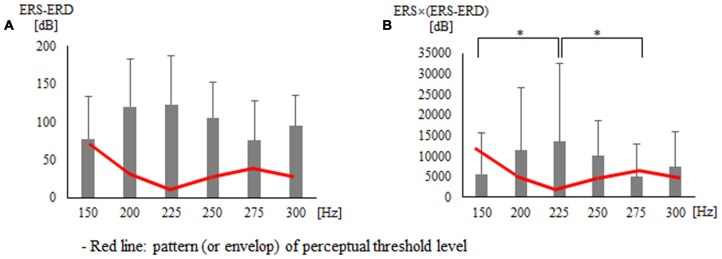
**Findings for two types of indices that presented significant differences in statistical analysis using the area of the ERD and ERS: (A**) ERS − ERD** (B)** ERS × (ERS − ERD). *p < 0.05.

Second, statistical analysis on indices using the area of ERD and ERS showed a significant difference for each vibration frequency with ERS − ERD (*p* = 0.033) and ERS × (ERS − ERD; *p* = 0.031; Table 2 and Figure 4). The *post hoc* verification revealed no significant difference for ERS − ERD (Figure 4A). ERS × (ERS − ERD) showed a significant difference between 150 Hz and 225 Hz (*p* = 0.016) and between 225 Hz and 275 Hz (*p* = 0.016; Figure 4B). The difference was greatest at 225 Hz. The association of perceptual threshold level with the two indices that showed a significant difference was examined and a significant difference was found in both ERS − ERD (*p* = 0.044) and ERS × (ERS − ERD; *p* = 0.050).

## Discussion

The objective of this study was to examine whether vibrotactile stimulation can be perceptually distinguished at high frequencies and quantified with ERD/ERS indices. The results showed a difference in the perceptual threshold level between 150 Hz and 200 Hz and between 150 Hz and 225 Hz for each frequency of the vibrotactile stimulation. These results correspond to those of Mountcastle et al. (1972), who suggested that humans are most sensitive to detection thresholds when the vibration frequency is 200–250 Hz. The sensitivity to frequency bands coincides with the sensitivity of Pacinian corpuscles. Thus, the perceptual distinction of vibrotactile stimulation at high frequencies may be dependent on the sensitive receptive field of Pacinian corpuscles.

On the other hand, when quantitatively distinguishing vibrotactile stimulations at different frequencies with specific indices utilizing ERD/ERS, no distinctive classification was found for each frequency either for ERD in the alpha domain or ERS in the beta domain, which are single indices. However, ERD of the alpha domain and ERS of the beta domain tended to increase at 200 and 225 Hz with a relatively low perceptual threshold (*p* = 0.075, *p* = 0.064). For ERD, which is mainly used as a dependent measurement value in the perceptual task, the magnitude increases when the given task is complicated or relatively more attention needs to be paid to perform the task (Boiten et al., 1992; Pfurtscheller and Klimesch, 1992; Dujardin et al., 1993). However, it is difficult to view vibrotactile stimulation in the present study as a complicated perceptual task because subjects passively perceived the presented vibrations, and no more attention was required for any concurrent additional task. Therefore, it is difficult to interpret that the increasing trend of the magnitude of vibrotactile stimulations in the range of 200–225 Hz was caused by the aforementioned factors. In contrast, considering that the alpha area of ERD was analyzed at 10–13 Hz, the ERD in the present study may be considered as a consequence of upper alpha, i.e., mu ERD. Considering the findings of Salmelin et al. (1995), who argued that an increase in the magnitude of mu ERD could be interpreted as a response in the primary somatosensory area, the increase in ERD of vibrotactile stimulation in the range of 200–225 Hz may be interpreted as a simple response to vibration itself. In addition, it is similar to the findings of an increase in contralateral ERD in response to 200 Hz tactile stimulation (He and Contreras-Vidal, 2015). This result suggests that the distinctions of vibrotactile stimulations at perceptual and physiological levels are identical. Moreover, ERS in the beta band is a measure of post-movement. Since it reflects a cumulative effect when neuronal activation increases, it is more likely to display an increase in magnitude for an entire palm vibrotactile stimulation than a digital one (Pfurtscheller and Zalaudek, 1998; Pfurtscheller and Lopes da Silva, 1999a). However, because the current experiment was conducted by exerting vibrotactile stimulation on the index finger, the ERS increase with a vibrotactile stimulation of 200–225 Hz cannot be attributed to the spatial aspect of neural associations alone but to the magnitude increase by the neural network during perceptual processing of the tactile information. Some studies have reported an ERS increase in the beta domain followed by continuous imagination regarding movement as well as post-movement (Pfurtscheller and Lopes da Silva, 1999b). Although the EEG findings of this study were limited to the C3 domain, the possibility of such an interpretation cannot fully be excluded considering the low spatial resolution; this represents one limitation of the present study.

On the other hand, there was a distinctive magnitude difference in vibrotactile stimulation at 150 Hz and 200 Hz that could be perceived with the (ERS − ERD) index, which subtracts ERD from ERS. Vibrotactile stimulations of 275 Hz and 300 Hz, which had a similar perceptual threshold as 200 Hz, also displayed an outstanding magnitude difference with vibrotactile stimulation of 200 Hz according to the (ERS − ERD) index. Because the (ERS − ERD) index displays the size of the total effect reflecting both excitatory and inhibitory effect followed by vibrotactile stimulation, it can be considered to calculate the amount of energy attributable to vibrotactile stimulation. From such a perspective, the ERS × (ERS − ERD) index, which amplifies the size effect of ERS considering the energy attributable to vibrotactile stimulation, clearly distinguishes the magnitude of the vibrotactile stimulation at 150 Hz and 225 Hz. The ERD × (ERS − ERD) index was found to clearly distinguish the magnitudes of the vibrotactile stimulation between 150 Hz and 200 Hz and between 200 Hz and 250 Hz. Extracting the common feature of all three indices not only clearly distinguished the vibrotactile stimulations of 150 Hz and 200–225 Hz at the perceptual threshold level but also markedly distinguished each vibrotactile stimulation to reflect the feature of the ERD/ERS single index, which only presented the inclination due to the small size of the effect. In other words, the above three indices can be used for quantitative measurement to clearly distinguish high vibration frequencies. To conclude, this study is significant in that it explored new indices based on ERD/ERS, while previous studies mainly examined the feature of ERD/ERS with regard to vibrotactile stimulations. In addition, a method was developed that enables more distinctive extraction of the feature of the ERD/ERS single index. However, the new index needs to be validated through future studies on vibrotactile stimulations.

## Author Contributions

M-HC and S-CC conceived and designed the experiments. M-HC and H-SK performed the experiments. M-HC and W-RK analyzed the data. H-SK and S-YG contributed reagents/materials/analysis tools. M-HC, BK and S-CC wrote the article. M-HC and BK drafted the article or revised it critically for important intellectual content.

## Conflict of Interest Statement

The authors declare that the research was conducted in the absence of any commercial or financial relationships that could be construed as a potential conflict of interest.
